# Chitosan-EDTA-Cellulose network as a green, recyclable and multifunctional biopolymeric organocatalyst for the one-pot synthesis of 2-amino-4*H*-pyran derivatives

**DOI:** 10.1038/s41598-022-10774-z

**Published:** 2022-05-23

**Authors:** Negin Rostami, Mohammad G. Dekamin, Ehsan Valiey, Hamidreza Fanimoghadam

**Affiliations:** Pharmaceutical and Biologically-Active Compounds Research Laboratory, Department of Chemistry, Iran University of Science and Technology, Tehran, 16846-13114 Iran

**Keywords:** Biocatalysis, Carbohydrates, Chemical modification, Environmental sciences, Catalysis, Chemical engineering, Environmental chemistry, Green chemistry, Medicinal chemistry, Organic chemistry, Supramolecular chemistry

## Abstract

In this research, cellulose grafted to chitosan by EDTA (Cs-EDTA-Cell) bio-based material is reported and characterized by a series of various methods and techniques such as FTIR, DRS-UV–Vis, TGA, FESEM, XRD and EDX analysis. In fact, the Cs-EDTA-Cell network is more thermally stable than pristine cellulose or chitosan. There is a plenty of both acidic and basic sites on the surface of this bio-based and biodegradable network, as a multifunctional organocatalyst, to proceed three-component synthesis of 2-amino-4*H*-pyran derivatives at room temperature in EtOH. The Cs-EDTA-Cell nanocatalyst can be easily recovered from the reaction mixture by using filtration and reused for at least five times without significant decrease in its catalytic activity. In general, the Cs-EDTA-Cell network, as a heterogeneous catalyst, demonstrated excellent catalytic activity in an environmentally-benign solvent to afford desired products in short reaction times and required simple experimental and work-up procedure compared to many protocols using similar catalytic systems.

## Introduction

Recently, nanoparticles have attracted considerable research interests due to their potential applications in catalysis science and technology in alignment with the principles of green chemistry^1,2^. Because of their nanoscale sizes and properties, they combine the advantages of both homogeneous and heterogeneous catalysts such as higher activity and selectivity as well as reuability^3,4^.

The use of environmentally benign, sustainable and efficient reusable catalysts has economic and environmental benefits^5,6^. One of the emerging green approaches for designing and applying heterogeneous catalytic systems is the use of biopolymers as appropriate biodegradable catalytic systems or supports^7–14^. In this regard, both cellulose^15^ and chitosan^16^, as economic and the most abundant biopolymers, have received considerable attention to be used in catalytic systems^17,18^ as well as drug delivery^19–21^, adsorbents^22–24^, hydrogels^25,26^, anti-bacterial wound dressing materials, etc^27,28^.

Chitosan is a linear polysaccharide with repeating unit β-(1 → 4) linked D-glucosamine and has a large number of amino and hydroxyl groups^29^. This biopolymer is obtained by chitin deacetylation in large scales^30^. Due to the presence of plenty of both amino and hydroxyl groups on the surface of chitosan polymer chains, chitosan itself can be used as a heterogeneous multifunctional organocatalyst for some organic transformations^31,32^. However, it is necessary sometimes to increase the catalytic activity of chitosan by grafting of appropriate functional groups^33–35^ or chelation of active metallic species^36^ as well as the combination of both strategies^37,38^. Furthermore, cellulose, as the most abundant biopolymer in nature, is composed of thousands of repeating β-(1 → 4) linked D-glucose units similar to the chitosan monomers^39^. Interestingly, the simultaneous use of chitosan and cellulose for heterogeneous catalytic systems provides higher thermal or mechanical materials at a lower cost^40^. Therefore, the preparation of new and more efficient chitosan–cellulose networks for different aforementioned applications is still in high demand.

On the other hand, multicomponent reactions (MCRs) have been considered as a superior synthetic strategy and an important subset of domino reactions in recent years. MCRs have received considerable attention from both academia and industry because of their various benefits over traditional multistep protocols in the synthesis of novel or complex heterocyclic scaffolds, especially in medicinal chemistry and pharmaceutical industry^41–43^. In addition, MCRs are valuable in organic synthesis because of their green chemical aspects such as high atom economy and low waste generation^44–46^. MCRs are highly convergent and require minimum time and effort to afford desired products. All of these merits can be intensified by using heterogeneous catalysts under mild reaction conditions^32,47–50^.

**Figure 1:**
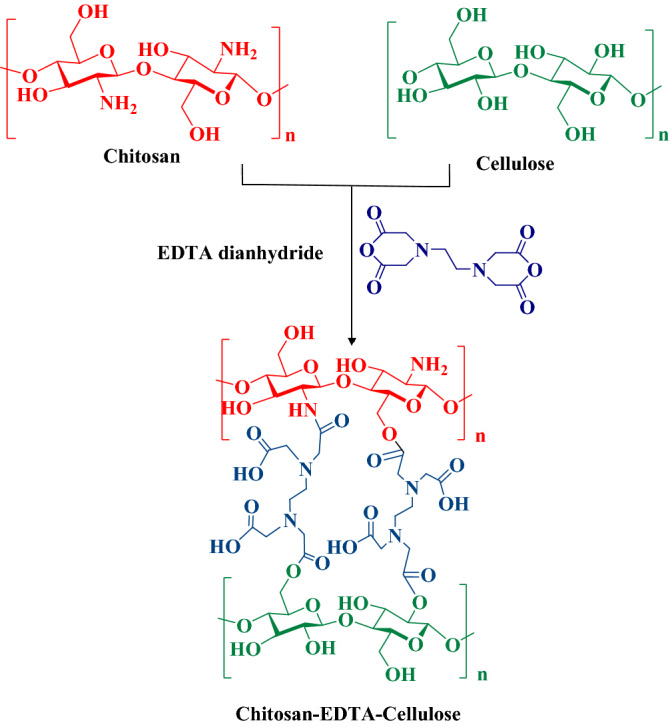
Schematic preparation of the multifunctional heterogeneous Cs-EDTA-Cell network (**1**).

One of the important biologically active scaffolds in medicinal chemistry is 2-amino-4*H*-pyran^51^. These compounds represent widespread pharmaceutical potential such as anticancer, anti-HIV, anti-inflammatory, antimalarial, antiviral and antihyperglycemic as well as DNA binder, cytotoxicity and insecticidal activities^52–54^. A simple and general protocol for preparation of 2-amino-4*H*-pyran derivatives involves a three-component one-pot cyclocondensation of ethyl acetoacetate, malononirilel/alkyl cyanoacetate and various carbonyl compounds. Several modified methods have been reported using different homogeneous or heterogeneous catalysts such as uera-chCl^55^, potassium phthalimide-*N*-oxyl^56^, potassium phthalimide under ball-milling^57^, Et_3_N under sonication^54^, CuFe_2_O_4_@starch^58^, CoFe_2_O_4_-Cell/Fe (III) SSZ^59^, KF-Al_2_O_3_^60^, Fe_3_O_4_/EDTA^61^, γ-Fe_2_O_3_-Im-Py)_2_WO_4_^62^, amine-functionalized SiO_2_@Fe_3_O_4_^63^, water extract of red mud^64^, sodium alginate^13,14^, isocyanurate-based periodic mesoporous organosilica^65^ and silver nanoparticles-decorated Preyssler functionalized cellulose^66^ in recent years. In continuation of our research group to expand the catalytic activity of pristine or modified biopolymers for different MCRs^10–14,32,67,68^, this report presents the use of Cs-EDTA-Cell as a biopolymer nanocatalyst for the synthesis of 2-amino-4*H*-pyran derivatives (Fig. 1).

## Results and discussion

### Characterization of the Cs-EDTA-Cell network (1)

**Figure 2:**
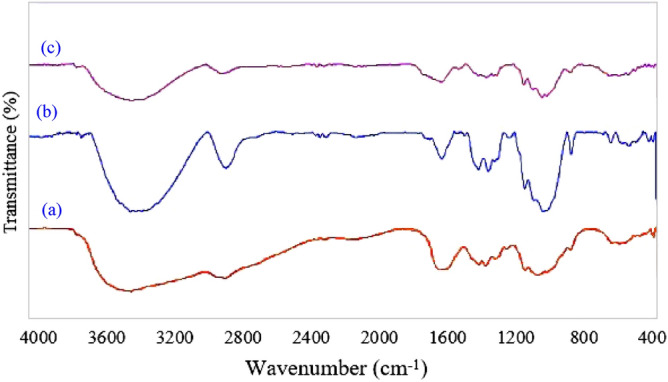
FTIR spectra of the chitosan (**a**), cellulose (**b**) and Cs-EDTA-Cell (**1**, c).

FTIR spectra of chitosan, cellulose and Cs-EDTA-Cell network (**1**) have been compared in Fig. 2. According to the FTIR spectra of chitosan (Fig. 2a) and cellulose (Fig. 2b), the wide band at ~ 3500–3200 cm^−1^ is related to intramolecular hydrogen bonds due to O–H stretching or N–H stretching vibrations. A peak found at ~ 2911 cm^−1^ is attributed to the C–H stretching of saccharide rings of both chitosan and cellulose. Furthermore, absorption band observed at 1660 cm^−1^ is assigned to the residual acetamide groups in the backbone of chitosan. On the other hand, The CH_2_ bending or CH_3_ symmetrical deformations of both chitosan and cellulose are appeared at ~ 1423–1375 cm^−1^. The asymmetric stretching of the C–O–C bridge were confirmed by the presence of bands at ~ 1150 cm^−1^. The broad signals at 1066 and 1028 cm^−1^ correspond to the C–O stretching. On the other hand, the FTIR spectra of Cs-EDTA-Cell network (**1**, Fig. 2c) show the absorption peaks at 1735 cm^−1^, 1685 cm^−1^, 1627 cm^−1^ corresponding to ester, acid and amide groups, respectively. All of these data demonstrate successful grafting of the cellulose to chitosan by using EDTA to form Cs-EDTA-Cell network (**1**).

**Figure 3:**
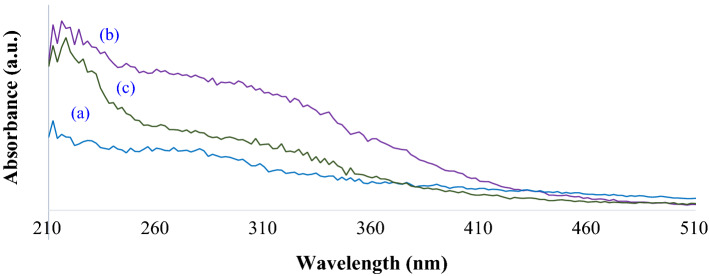
DRUV spectra of the chitosan (**a**), cellulose (**b**) and Cs-EDTA-Cell (**1**, c).

Figure 3 shows the diffuse reflectance UV–Visible (DRUV) spectra of chitosan, cellulose and Cs-EDTA-Cell network (**1**), respectively. In fact, chitosan and especially cellulose show very simple DRUV spectra. Hence, the characteristic absorption near 220 nm correspond to the pristine chitosan (Fig. 3a–b). The DRUV absorption spectra of pure chitosan and cellulose exhibited maximum absorption peak (λ_max_) around 220 nm. Furthermore, EDTA dianhydride cross-links chitosan and cellulose backbones and forms new amide and ester functional groups on reaction with amine groups of chitosan or the hydroxyl groups on both chitosan and cellulose. Consequently, the λ_max_ in Cs-EDTA-Cell network (**1**) shifted to 230 nm due to formation of the new amide and esteric functional groups.

**Figure 4:**
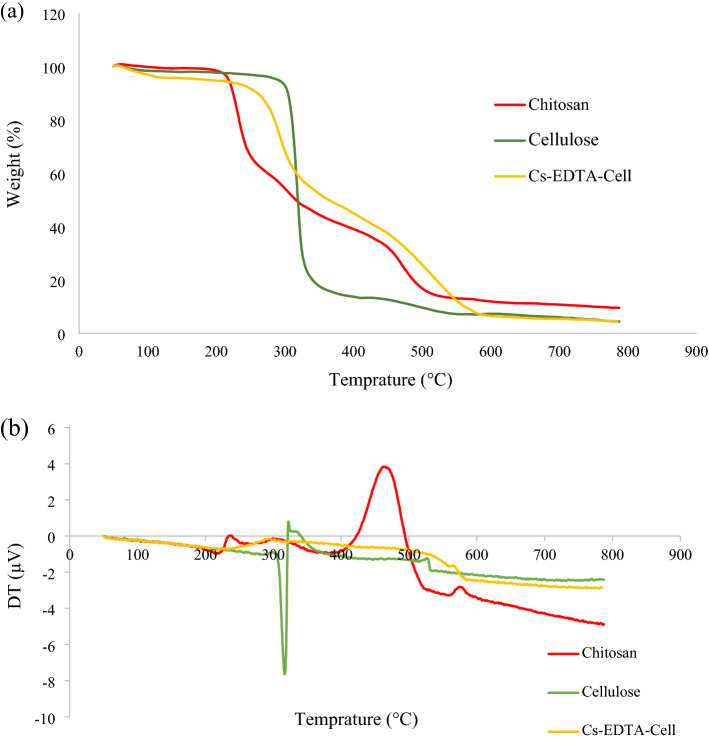
TGA (**a**) and DTA (**b**) curves of chitosan, cellulose and Cs-EDTA-Cell network (**1**).

Thermal stability of the bio-based Cs-EDTA-Cell network (**1**) was evaluated under air atmosphere at the range of 50–800 °C and a rate of 5 °C/min compared to chitosan and cellulose (Fig. 4). The TGA curves of the Cs-EDTA-Cell network and cellulose showed that their total weight losses were around 95%. In fact, commercial chitosan is less thermally stable than cellulose and demonstrates a two-stage weight losses starting at 200 and 290 °C. On the other hand, cellulose demonstrate higher thermal stability than chitosan and its decomposition starts at about 285 °C. However, the most of weight loss of cellulose (86%) occurs up to 405 °C in contrast to chitosan, which about of 32% of its mass has remained at this temperature. Interestingly, Cs-EDTA-Cell network (**1**) was found to be more thermally stable than its moieties, especially commercial chitosan^68^ and cellulose^69^. Indeed, the most mass loss of the Cs-EDTA-Cell network (**1**, 56%) was occurred at about 220–405 °C that can be attributed to the decomposition of both chitosan and cellulose moieties as well as EDTA linker. This characteristic is very important in designing and application of reusable heterogeneous catalytic systems, which require recycling and subsequent consecutive heating during catalytic cycles or thermal reactivation.

**Figure 5:**
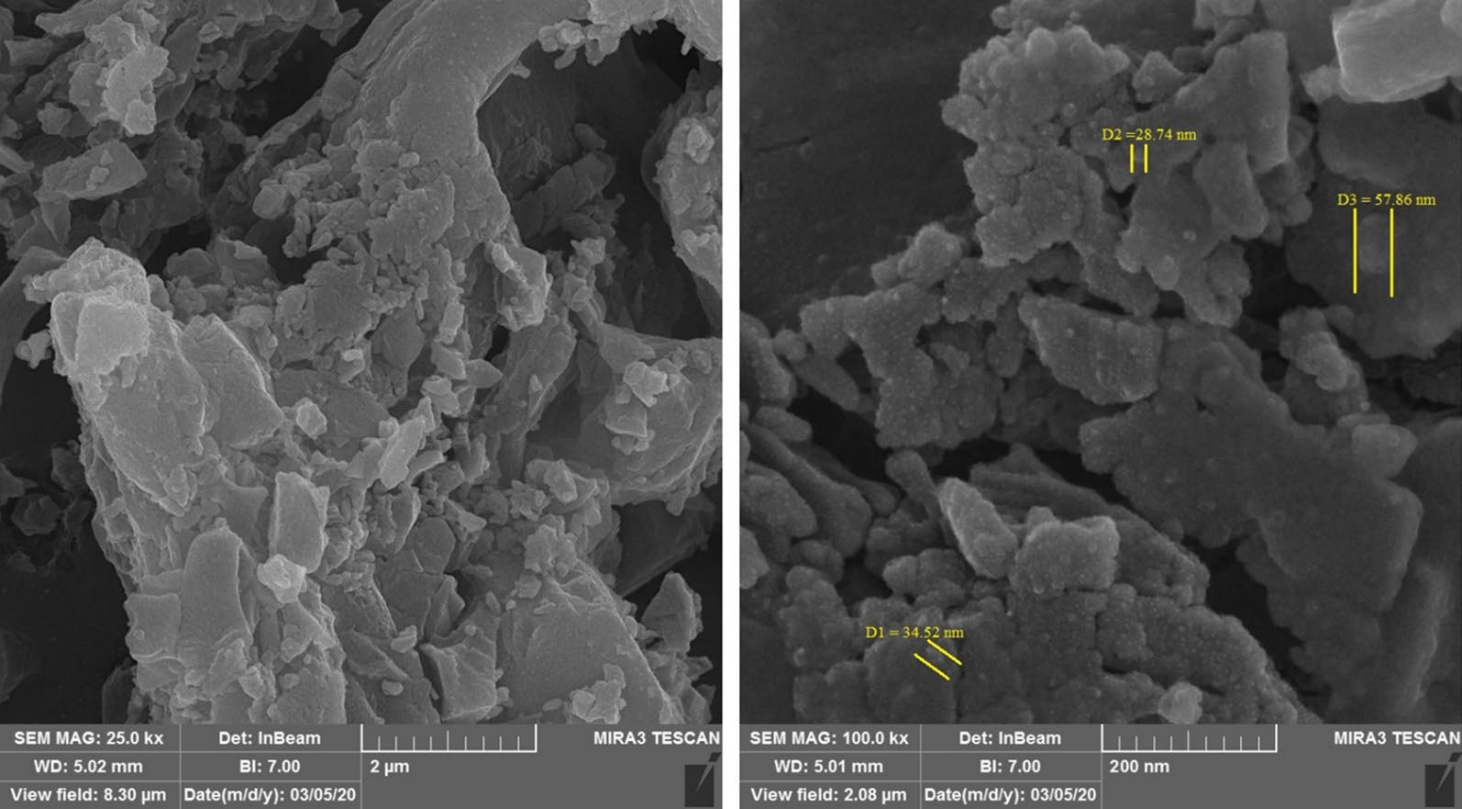
FESEM images of the Cs-EDTA-Cell network (**1**).

The surface morphologies of the Cs-EDTA-Cell network (**1**) determined by FESEM analysis have been shown in Fig. 5. Indeed, pure chitosan^70^ and cellulose^71^ have smooth and layered surface morphologies according to the literature data. By comparing of the obtained FESEM images with surface morphologies of both pure chitosan and cellulose, it can be concluded that chitosan and cellulose are successfully grafted together by the EDTA linker. The average size of the spherical particles was measured to be about 28–57 nm in the Cs-EDTA-Cell network (**1**).

**Figure 6:**
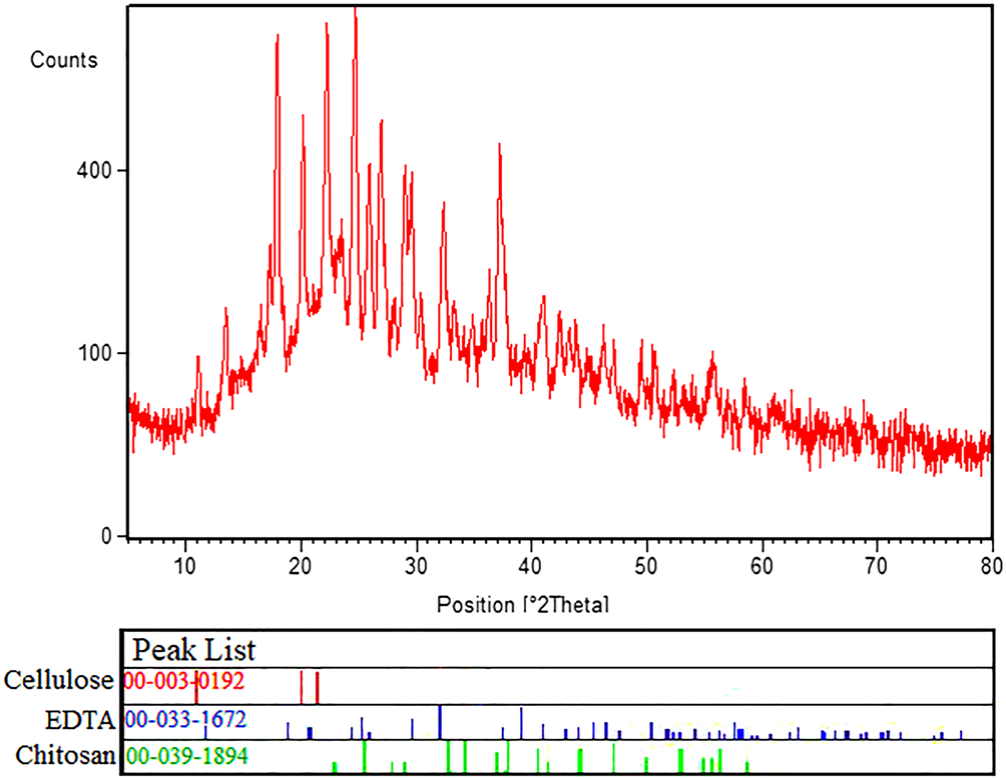
XRD pattern of the Cs-EDTA-Cell network (**1**).

The XRD pattern of Cs-EDTA-Cell network (**1**) was compared to chitosan, cellulose and EDTA reference cards (Fig. 6). Sharp peaks in the XRD pattern indicate the presence of crystalline regions in the network. According to the obtained results, all the diffraction angles (2θ = 10.0, 11.0, 18.0, 20.0, 21.3, 24.8, 26.0, 27.0, 29.0, 29.5, 32.4, 37.2 and 41.0) correspond to the standard XRD pattern of chitosan (JCPDS card No. 00-039-1894), cellulose (JCPDS card No. 00-003-0192) and EDTA (JCPDS card No. 00-033-1672). These data demonstrate that cellulose was grafted successfully to the chitosan backbone by EDTA as a bidentate cross-linker.

**Figure 7:**
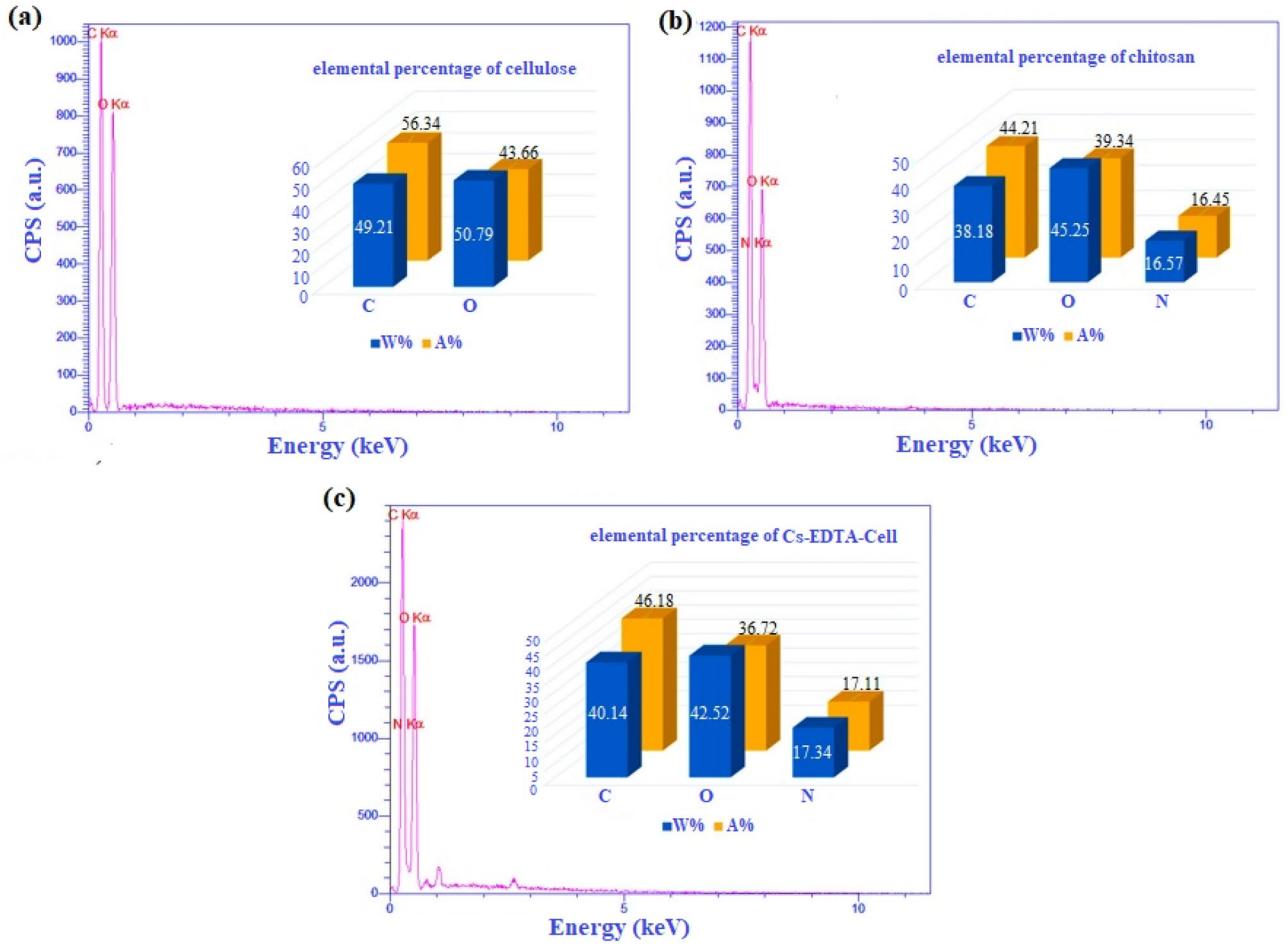
Energy-dispersive X-ray (EDX) spectroscopy pattern of cellulose (**a**), chitosan (**b**) and Cs-EDTA-Cell network (**1**, **c**).

The EDX spectra and elemental charts of cellulose, chitosan and Cs-EDTA-Cell network (**1**) excluding the presence of hydrogen atoms are presented in Fig. 7. Cellulose and chitosan are comprised of C, O and C, N, O, respectively (Fig. 7a–b). Indeed, EDX analysis of Cs-EDTA-Cell network (**1**) confirmed clearly the presence of C, N and O atoms (Fig. 7c). As can be seen from the obtained results by the EDX spectra, the presence of C, O and N peaks can be ascribed to the structure of chitosan and cellulose biopolymers as well as EDTA. Since EDTA contains two nitrogen atoms in its structure, increasing of the percentage of N in the Cs-EDTA-Cell network (**1**) indicates that cellulose has been successfully grafted to chitosan by the EDTA linker.

### Evaluation of the catalytic activity of Cs-EDTA-Cell network (1) for the synthesis of 2‐amino‐3-cyano-4*H*‐pyran derivatives 5a-l

After characterization of the prepared Cs-EDTA-Cell network (**1**), it was investigated in the one-pot three-component synthesis of 2‐amino‐3-cyano-4*H*‐pyran derivatives as a heterogeneous catalyst. First, to find the optimized conditions, the reaction of ethyl acetoacetate (**2**), 4-chlorobenzaldehyde (**3a**), malononitrile (**4**) was selected as the model reaction. The effect of various parameters such as the amount of catalyst loading, solvent, temperature, and time on the model reaction was examined. The obtained results is shown in Table 1. In the absence of any catalyst and under solvent-free conditions at 100 °C, only a trace amount of product ethyl 6-amino-4-(4-chlorophenyl)-5-cyano-2-methyl-4*H*-pyran-3-carboxylate (**5a**) was obtained after two hours (Table 1, entry 1). On the other hand, in the presence of 10 mg of chitosan, cellulose and EDTA the obtained yield was only 40, 35 and 65% in EtOH, respectively (Table 1, entries 2–4). These experiments showed that the reaction needs a more effective catalyst. For this purpose, the model reaction was carried out in the presence of Cs-EDTA-Cell network (**1**) in various solvents at different temperatures (Table 1, entries 5–12). Interestingly, the best result was obtained when 0.01 g of Cs-EDTA-Cell was used in ETOH at room temperature (entry 5). In next experiments, the model reaction was investigated in the presence of higher or lower catalyst loadings (Table 1, entries 13, 14). Hence, 0.01 g of Cs-EDTA-Cell loading in ETOH at room temperature was selected as the optimized reaction conditions and used in further experiments to develop the scope of this protocol for the synthesis of other derivatives of 2‐amino‐3-cyano-4*H*‐pyran scaffold **5a-l**.

**Table 1:** Optimizing of different parameters for the model reaction in the synthesis of **5a** catalyzed by Cs-EDTA-Cell network (**1**)^a^.


Entry	Catalyst	Catalyst loading (g)	Solvent	Temp (°C)	Time (min)	Yield^b^ 5a (%)
1	–	–	–	100	120	< 5
2	Chitosan	0.01	EtOH	Reflux	20	40
3	Cellulose	0.01	EtOH	Reflux	20	35
4	EDTA	0.01	EtOH	Reflux	20	65
5	Cs-EDTA-Cell	0.01	EtOH	rt	10	96
6	Cs-EDTA-Cell	0.01	EtOH	Reflux	10	96
7	Cs-EDTA-Cell	0.01	H_2_O	rt	90	37
8	Cs-EDTA-Cell	0.01	H_2_O	Reflux	60	65
9	Cs-EDTA-Cell	0.01	EtOH/H_2_O (1:1)	rt	60	70
10	Cs-EDTA-Cell	0.01	EtOH/H_2_O (1:1)	78	30	75
11	Cs-EDTA-Cell	0.01	Toluene	Reflux	20	40
12	Cs-EDTA-Cell	0.01	MeCN	Reflux	20	45
13	Cs-EDTA-Cell	0.02	EtOH	Reflux	10	96
14	Cs-EDTA-Cell	0.003	EtOH	Reflux	20	65

^a^Reaction conditions: ethyl acetoacetate (**2**, 1.0 mmol), aldehyde (**3a**, 1.0 mmol) and malononirile (**4**, 1.0 mmol) in the presence of Cs-EDTA-Cell network (**1**) was added to the solvent (3.0 ml) unless otherwise stated.

^b^Isolated yields.

The required time for other aromatic and heteroaromatic derivatives of aldehydes **4a-l** when Cs-EDTA-Cell nanomaterial (**1**) was employed, as a heterogeneous organocatalyst, under optimized conditions are summarized in Table 2. All studied aldehydes **4a-l** were smoothly involved in the optimized conditions to afford corresponding 2‐amino‐3-cyano-4*H*‐pyran scaffold **5a-l**. In addition, aldehydes bearing electron-withdrawing groups (entries 1–7) generally afforded the desired products in higher yields and short reaction times compared to benzaldehyde or aldehydes having electron-withdrawing groups (entries 8–12).

**Table 2:** Scope of the synthesis of 2‐amino‐3-cyano-4*H*‐pyran derivatives **5a-l** catalyzed by Cs-EDTA-Cell (**1**) under optimized reaction conditions^a^.


Entry	Ar	Product^b^	Time (min)	Yield^c^ (%)	M.p. (°C)	M.p. (°C) (Lit.)	Ref
1	4-ClC_6_H_4_	**5a**	10	96	170–171	171–172	^72^
2	2-ClC_6_H_4_	**5b**	20	94	180–181	179–181	^72^
3	4-NO_2_C_6_H_4_	**5c**	15	92	173–175	175–176	^72^
4	3-NO_2_C_6_H_4_	**5d**	15	91	180–182	182–184	^73^
5	2-FC_6_H_4_	**5e**	20	85	159–161	158–160	^74^
6	4-BrC_6_H_4_	**5f.**	25	93	171–173	172–173	^75^
7	3-Pyridine	**5j**	25	90	178–179	178–180	^76^
8	C_6_H_5_	**5 g**	20	85	175–177	177	^77^
9	3-HOC_6_H_4_	**5 h**	25	90	165–167	164–166	^55^
10	3-MeOC_6_H_4_	**5i**	25	91	121–123	122–124	^74^
11	2-Thiophene	**5 k**	25	89	174–176	170–172	^78^
12	2-Furan	**5 l**	25	90	172–174	171–172	^79^

^a^Reaction conditions: ethyl acetoacetate (**2**, 1.0 mmol), aldehyde (**3a-l**, 1.0 mmol) and malononirile (**4**, 1.0 mmol) in the presence of Cs-EDTA-Cell network (**1**, 0.01 g) was added to the EtOH (3.0 ml) at room temperature.

^b^All compounds are known and their structures were established from their spectral data and melting points as compared with literature values.

^c^Yields refer to the isolated products.

### Mechanistic aspects of the synthesis of 2‐amino‐3-cyano-4*H*‐pyran derivatives (5a-l) catalyzed by Cs-EDTA-Cell network (1)

**Figure 8:**
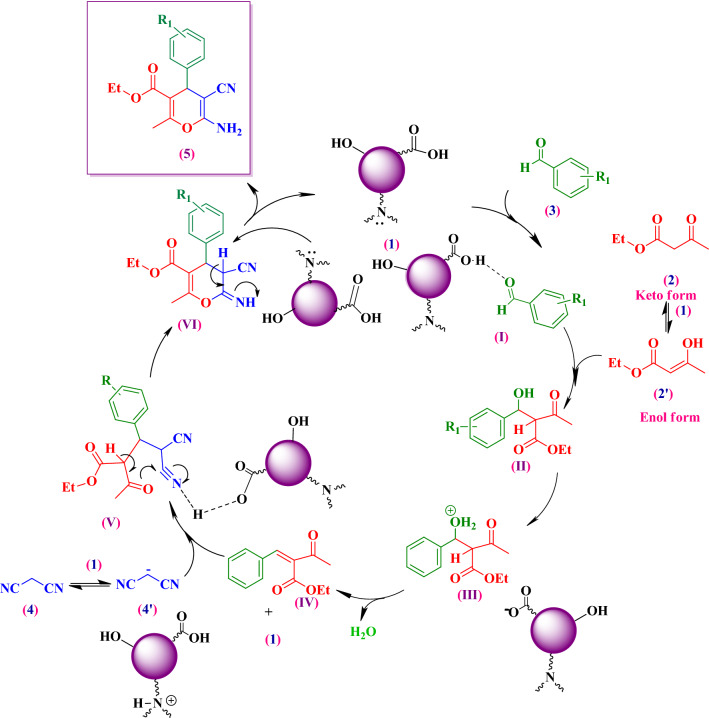
Proposed mechanism for the one-pot synthesis of **5a-l** catalyzed by the multifunctional Cs-EDTA-Cell network organocatalyst (**1**).

A plausible mechanism has been proposed for the reaction of ethyl acetoacetate (**2**), aromatic aldehydes (**3a-l**) and malononirile (**4**) in the presence of Cs-EDTA-Cell network (**1**, Fig. 8). According to this mechanism, both carboxylic acid and hydroxyl functional groups as well as amine basic sites of the multifunctional organocatalyst **1** with proper geometry are the main active sites that influence and proceed different reaction steps. On the other hand, the by-product of the reaction is water molecules, which they can be absorbed by the Cs-EDTA-Cell network (**1**) efficiently in the EtOH solvent and accelerate the reaction.

### Investigating of the reusability of Cs-EDTA-Cell network (1) in the synthesis of 2‐amino‐3-cyano-4*H*‐pyran derivative 5a

**Figure 9:**
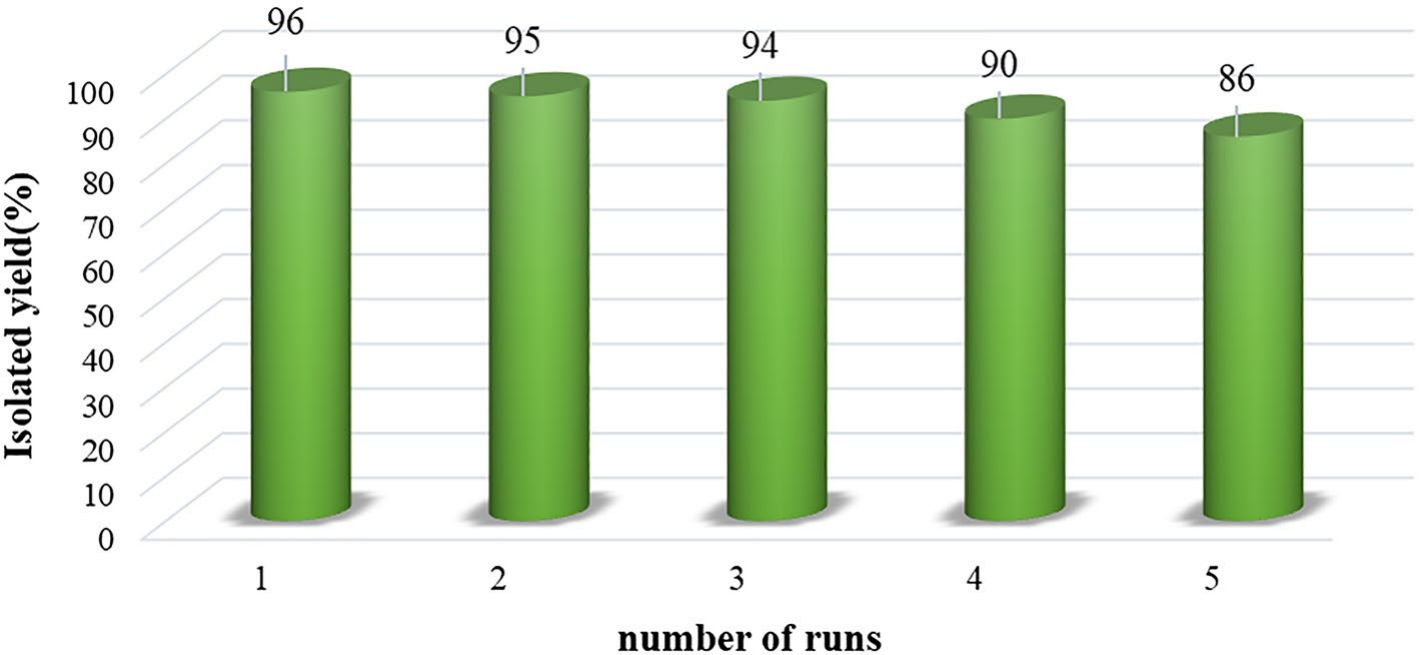
Reusability of the multifunctional heterogeneous Cs-EDTA-Cell network (**1**) in five consecutive runs for the synthesis of **5a**.

Finally, the recyclability of the catalyst **1** was examined under the optimized conditions. The results are shown in Fig. 9. Consecutive experiments for the model reaction between ethyl acetoacetate (**2**), 4-chlorobenzaldehyde (**3a**) and malononirile (**4**) under the optimized conditions were run and the recycled catalyst was used for the next experiment after activation. As shown in Fig. 9, the catalyst was recycled five times with only 10% loss in the model reaction yield. These data demonstrate proper stability of the heterogeneous Cs-EDTA-Cell catalyst (**1**) for the synthesis of 2‐amino‐3-cyano-4*H*‐pyran derivatives **5**.

### Comparison of the catalytic activity of Cs-EDTA-Cell network (1) for the synthesis of 2‐amino‐3-cyano-4*H*‐pyran derivatives 5a with other catalytic systems

To show the merits of the synthesis of 2‐amino‐3-cyano-4*H*‐pyran derivatives catalyzed by Cs-EDTA-Cell network (**1**), its catalytic performance, as a heterogeneous multifunctional catalyst, has been compared with the previous catalytic systems for preparation of **5a**. The results have been summarized in Table 3. Comparison of the results shows that the catalytic activity of Cs-EDTA-Cell network (**1**) is superior to the most of introduced protocols in terms of catalyst loading, obtained yields, simplicity of the catalyst preparation process, and required temperature and time.

**Table 3:** Comparative results of the catalytic activity of Cs-EDTA-Cell network (**1**) for the synthesis of **5a.**

Entry	Catalyst	Catalyst loading (mg)	Solvent	Temp. (°C)	Time (min)	Yield (%)	Ref
1	Sodium alginate	20	EtOH	Reflux	195	84	^14^
2	Uera-ChCl	30	DES	80	120	88	^55^
3	CuFe_2_O_4_@starch	30	EtOH	rt	20	96	^58^
4	CoFe_2_O_4_-Cell/Fe (III) SSZ	160	EtOH	60	8	98	^59^
5	KF-Al_2_O_3_	16 mg	EtOH	rt	180	91	^60^
6	Fe_3_O_4_/EDTA	5 mg	EtOH	rt	13	95	^61^
7	Cs-EDTA-Cell	10 mg	EtOH	rt	10	96	This work

## Conclusions

In this study, we designed and developed the thermally stable Cs-EDTA-Cell network, as a heterogeneous multifunctional organocatlyst, based on the most abundant natural biopolymers including chitosan and cellulose using the EDTA cross-linker. The Cs-EDTA-Cell network was prepared in one-pot and simple procedure and then characterized adequately by various microscopic or spectroscopic methods as well as analytical techniques. The new Cs-EDTA-Cell catalyst was investigated in the synthesis of 2-amino-4*H*-pyran derivatives at room temperature via a one-pot, multicomponent reaction between ethyl acetoacetate aromatic aldehydes, and malononirile in EtOH as a green solvent. Several advantages of this protocol are broad scope of substrates using low catalyst loading, high to excellent yields of the desired products, and short reaction times as well as simple work-up procedure and reusability of the catalyst for at least five consecutive runs.

## Experimental section

### Materials and methods

Chitosan (MW = 190–310 kDa, medium molecular weight, 75–85% deacetylated, supplied by Across company), cellulose microcrystalline for thin-layer chromatography (provided by Merck company) and ethylenediaminetetraacetic acid (EDTA, MW = 292.24 g.mol^−1^) were used for preparation of Cs-EDTA-Cell network (**1**). Ethyl acetoacetate, malononitrile and a wide range of aldehydes were purchased from the international chemical companies including Merck or Sigma-Aldrich. The chemicals were used as received except for benzaldehyde, which a fresh sample of it was distilled. Melting points of the desired products were measured on an Electrothermal 9100 apparatus and are uncorrected. The functional groups of the samples were identified by FTIR spectroscopy on a Shimadzu FTIR -8400S spectrometer in the range of 400–4000 cm^−1^ using KBr discs. The DRUV spectroscopy of samples was performed by a Shimadzu UV-2550 spectrometer. The morphology of the nanocatalyst was observed by FESEM TESCAN-MIRA3. TGA and DTG curves of the Cs-EDTA-Cell network (**1**) were recorded by Bahr company STA 504. X-ray diffraction (XRD) pattern was taken by the Bruker D8 Advance device. Composition of the catalyst was determined by Energy-dispersive X-ray (EDX) spectroscopy using a Numerix DXP-X10P instrument. ^1^H NMR spectra of the isolated products were recorded at 500 MHz using Varian-INOVA spectrometer. The analytical TLC experiments were accomplished by using Merck Kieselgel 60 F-254 Al-plates and then visualized by UV light and iodine vapor.

### Preparation of the bio-based Cs-EDTA-Cell network (*1*)

Cellulose (1.0 g) was dissolved in NaOH (1 M, 8.0 ml) in a 50 ml round-bottomed flask under Ar atmosphere at 80 °C for 3 h. Then, chitosan (1.0 g) was added to the reaction mixture and stirred for one hour. After that, EDTA dianhydride (cross-linker, 8.0 g) -prepared according to Tülü and Geckeler procedure^80^- was added and the mixtures was stirred for 12 h at 80 °C. Then, HCl solution (1.0 M) was added dropwise to adjust pH around 7.0. Eventually, the white solid powder was filtered using a vacuum pump and washed several times with distilled water and Et_2_O and then, dried in an oven at 50 °C for 4 h^81^.

### Determination of the acidity of Cs-EDTA-Cell network (*1*)

The acidity of the Cs-EDTA-Cell catalyst (**1**) was calculated by the back-titration. In this procedure, the Cs-EDTA-Cell (0.1 g), NaCl (0.1 g) and NaOH (0.1 M, 2 ml) were added to 7 mL of distilled water and stirred at room temperature for 24 h. During this time, all acidic protons, which released from the Cs-EDTA-Cell network (**1**) were completely neutralized by the hydroxide ions of NaOH. Then, two drops of the phenolphthalein indicator aqueous solution were added to the mixture and the color of the solution changed to pink. Eventually, the excess of hydroxide ions was titrated using HCl solution (0.1 M, 1.11 ml) to turn the color of the obtained mixture into colorless. This point indicates the neutral PH and demonstrates that the acidity of Cs-EDTA-Cell (**1**) is about 0.89 mmol.g^-1^.

### General procedure for the synthesis of 2-amino-4H-pyran *5a-l* derivatives catalyzed by Cs-EDTA-Cell network (*1*)

In a 5 ml round-bottomed flask, a mixture of ethyl acetoacetate (**2**, 1 mmol), aromatic aldehyde (**3**, 1 mmol), malononitrile (**4**, 1 mmol) and Cs-EDTA-Cell (**1**, 0.01 g) were added to EtOH (3 ml) in a 25 ml round-bottomed flask. The obtained mixture was mechanically stirred at room temperature for appropriate time indicated in Table 2. After completion of the reaction, which was confirmed by thin layer chromatography (TLC), hot EtOH (5 ml) was added and stirring continued at room temperature for 10 min. The catalyst **1** was separated easily by filtration and the filtrate was allowed at room temperature to precipitate pure product. The recycled catalyst **1** was kept in an oven at 50 °C for one hour and then reused for the next runs.

### Physical and spectral data for the selected compounds *5a*, *5c*, *5j* and *5 k*

Ethyl 6-amino-4-(4-chlorophenyl)-5-cyano-2-methyl-4*H*-pyran-3-carboxylate (**5a**): mp = 170–171 °C; Yield = 96%; ^1^H NMR (500 MHz, DMSO-*d*_*6*_), *δ (*ppm): 7.34 (2H, t, *J* = 8.4 Hz, Ar–H), 7.14 (2H, t, *J* = 8.4 Hz, Ar–H), 6.89 (2H, brs, NH_2_), 4.29 (1H, s, CH), 3.96 (2H, q, *J* = 7.2 Hz, CH_2_), 2.28 (3H, s, CH_3_), 1.01 (3H, t, *J* = 7.2 Hz, CH_3_).

Ethyl 6-amino-5-cyano-2-methyl-4-(4-nitrophenyl)-4*H*-pyran-3-carboxylate (**5c**): mp = 173–175 °C, Yield = 92%;^1^H NMR (500 MHz, DMSO-*d*_*6*_), *δ* (ppm): 8.16 (2H, d, *J* = 8.7 Hz, Ar–H), 7.40 (2H, d, *J* = 8.7 Hz, Ar–H), 7.05 (2H, brs, NH_2_), 4.44 (1H, s, CH), 3.92 (2H, q, *J* = 7.2 Hz, CH_2_), 2.33 (3H, s, CH_3_), 0.98 (3H, t, *J* = 7.2 Hz, CH_3_).

Ethyl 6-amino-5-cyano-2-methyl-4-(pyridin-3-yl)-4*H*-pyran-3-carboxylate (**5j**): mp = 178–179 °C; Yield = 90%; ^1^H NMR (500 MHz, DMSO-*d*_*6*_), *δ* (ppm): 8.44 (1H, s, Ar–H), 8.41 (1H, d, *J* = 1.8 Hz, Ar–H), 7.56 (1H, d, *J* = 6.9 Hz, Ar–H), 7.36 (1H, dd, *J* = 7.8, 4.8 Hz, Ar), 7.01 (2H, brs, NH_2_), 4.35 (1H, s, CH), 3.96 (2H, *J* = 7.2 Hz, CH_2_), 2.33 (3H, s, CH_3_), 1.01 (3H, t, *J* = 7.2 Hz, CH_3_).

Ethyl 6-amino-5-cyano-2-methyl-4-(thiophen-2-yl)-4*H*-pyran-3-carboxylate (**5 k**): mp = 174–176 °C; Yield = 89%; ^1^H NMR (500 MHz, DMSO-*d*_*6*_), *δ* (ppm): 7.35 (1H, d, *J* = 4.8 Hz, Ar–H), 7.02 (2H, brs, NH_2_), 6.93 (1H, t, *J* = 4.2 Hz, Ar–H), 6.83 (1H, d, *J* = 3.0 Hz, Ar–H), 4.63 (1H, s, CH), 4.07 (2H, *J* = 7.2 Hz, CH_2_), 2.27 (3H, s, CH_3_), 1.14 (3H, t, *J* = 7.2 Hz, CH_3_).

## Supplementary Information

Supplementary Information.
