# Supplementary material for: Chitosan-EDTA-Cellulose network as a green, recyclable and multifunctional biopolymeric organocatalyst for the one-pot synthesis of 2-amino-4H-pyran derivatives
Source: Sci Rep. 2022 May 23;12:8642. doi: 10.1038/s41598-022-10774-z (PMC9126885; doi:10.1038/s41598-022-10774-z)
Supplement: Supplementary file 1 — Supplementary Information. [file 41598_2022_10774_MOESM1_ESM.docx]

**Electronic Supporting Information**

**Chitosan-EDTA-Cellulose network as a green, recyclable and multifunctional biopolymeric organocatalyst for the one-pot synthesis of** **2-amino-4*H*-pyran derivatives**

Negin Rostami^a^, Mohammad G. Dekamin^a,^*, Ehsan Valiey^a^ and Hamidreza Fanimoghadam^a^

*^a^ Pharmaceutical and Biologically-Active Compounds Research Laboratory, Department of Chemistry, Iran University of Science and Technology, Tehran 16846-13114, Iran*

**Corresponding author. E-mail: mdekamin@iust.ac.ir; Tel: +98-21-77 240 284; Fax: +98-21-7730*

| **Page** | **Content** |
| --- | --- |
| S1 | Title page |
| S2 | Physical and spectral data for the selected compounds **5a**, **5c**, **5j** and **5k** |
| S2 | Chemical characterization of Ethyl 6-amino-4-(4-chlorophenyl)-5-cyano-2-methyl-4*H*-pyran-3-carboxylate (**5a**) |
| S3 | Chemical characterization of Ethyl 6-amino-5-cyano-2-methyl-4-(4-nitrophenyl)-4*H*-pyran-3-carboxylate (**5c**) |
| S4 | Chemical characterization of Ethyl 6-amino-5-cyano-2-methyl-4-(pyridin-3-yl)-4*H*-pyran-3-carboxylate (**5j**) |
| S5 | Chemical characterization of Ethyl 6-amino-5-cyano-2-methyl-4-(thiophen-2-yl)-4*H*-pyran-3-carboxylate (**5k**) |

**Physical and spectral data for the selected compounds 5a, 5c, 5j and 5k:**

1) **Ethyl 6-amino-4-(4-chlorophenyl)-5-cyano-2-methyl-4*H*-pyran-3-carboxylate (5a)**: mp = 170 − 171 °C; Yield = 96 %; ^1^H NMR (500 MHz, DMSO‑*d_6_*), *δ (*ppm): 7.34 (2H, t, *J* = 8.4 Hz, Ar-H), 7.14 (2H, t, *J* = 8.4 Hz, Ar-H), 6.89 (2H, brs, NH_2_), 4.29 (1H, s, CH), 3.96 (2H, q, *J* = 7.2 Hz, CH_2_), 2.28 (3H, s, CH_3_), 1.01 (3H, t, *J* = 7.2 Hz, CH_3_).


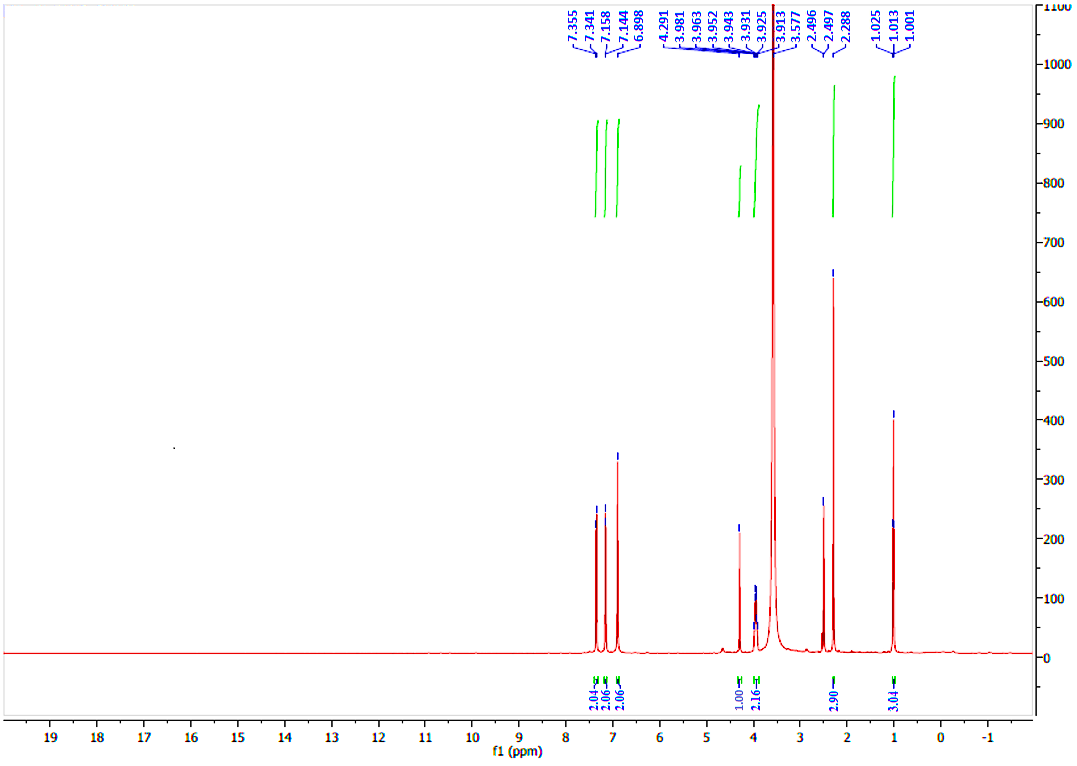


**Fig. 8.** ^1^H NMR spectrum of ethyl 6-amino-4-(4-chlorophenyl)-5-cyano-2-methyl-4*H*-pyran-3-carboxylate (**5a**).

2) **Ethyl 6-amino-5-cyano-2-methyl-4-(4-nitrophenyl)-4H-pyran-3-carboxylate (5c)**: mp = 173 − 175 °C, Yield = 92 %;^1^H NMR (500 MHz, DMSO‑d_6_), δ (ppm): 8.16 (2H, d, J = 8.7 Hz, Ar-H), 7.40 (2H, d, J = 8.7 Hz, Ar-H), 7.05 (2H, brs, NH_2_), 4.44 (1H, s, CH), 3.92 (2H, q, J = 7.2 Hz, CH_2_), 2.33 (3H, s, CH_3_), 0.98 (3H, t, J = 7.2 Hz, CH_3_).


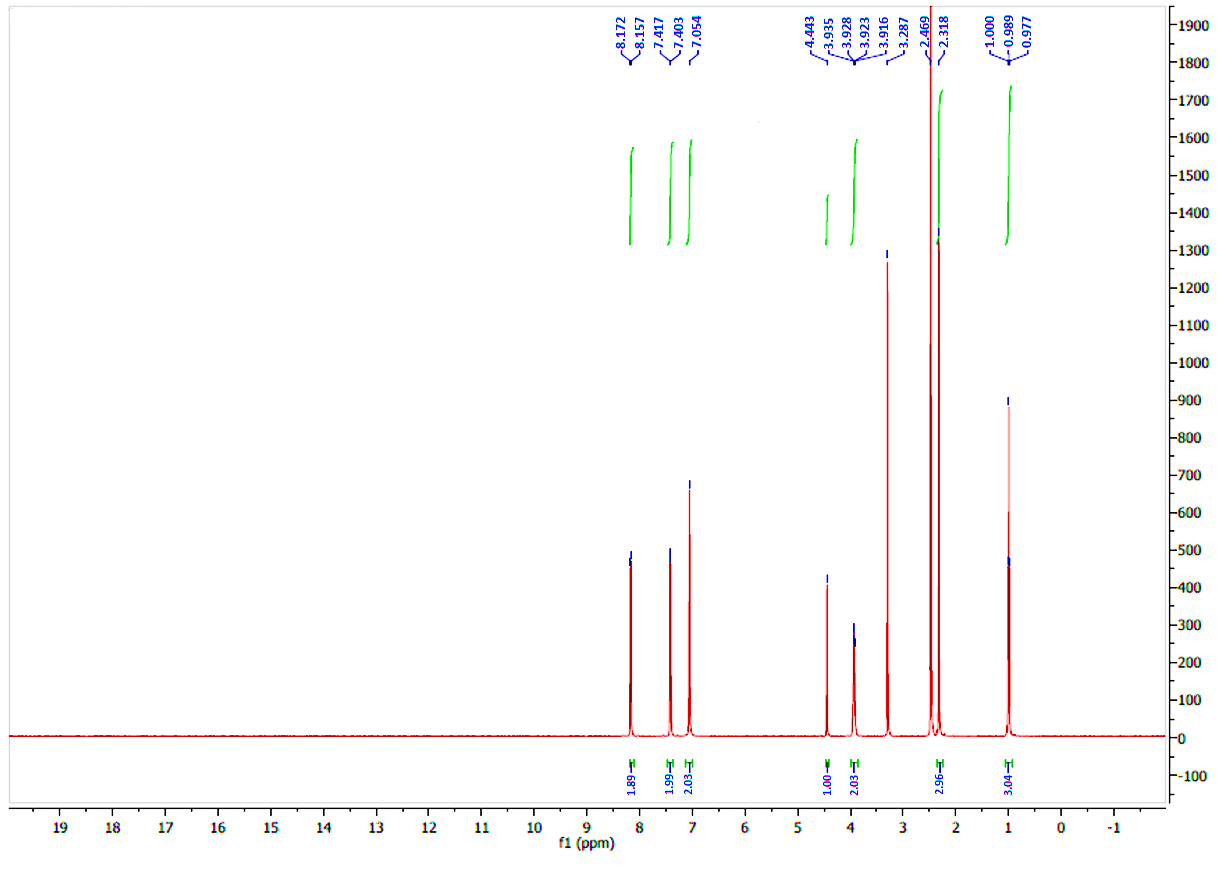


**Fig. 9.** ^1^H NMR spectrum of ethyl 6-amino-5-cyano-2-methyl-4-(4-nitrophenyl)-4*H*-pyran-3-carboxylate (**5c**).

3) **Ethyl 6-amino-5-cyano-2-methyl-4-(pyridin-3-yl)-4H-pyran-3-carboxylate (5j)**: mp = 178 − 179 °C; Yield = 90 %; ^1^H NMR (500 MHz, DMSO‑d_6_), δ (ppm): 8.44 (1H, s, Ar-H), 8.41 (1H, d, J = 1.8 Hz, Ar-H), 7.56 (1H, d, J = 6.9 Hz, Ar-H), 7.36 (1H, dd, J = 7.8, 4.8 Hz, Ar), 7.01 (2H, brs, NH_2_), 4.35 (1H, s, CH), 3.96 (2H, *J* = 7.2 Hz, CH_2_), 2.33 (3H, s, CH_3_), 1.01 (3H, t, J = 7.2 Hz, CH_3_).


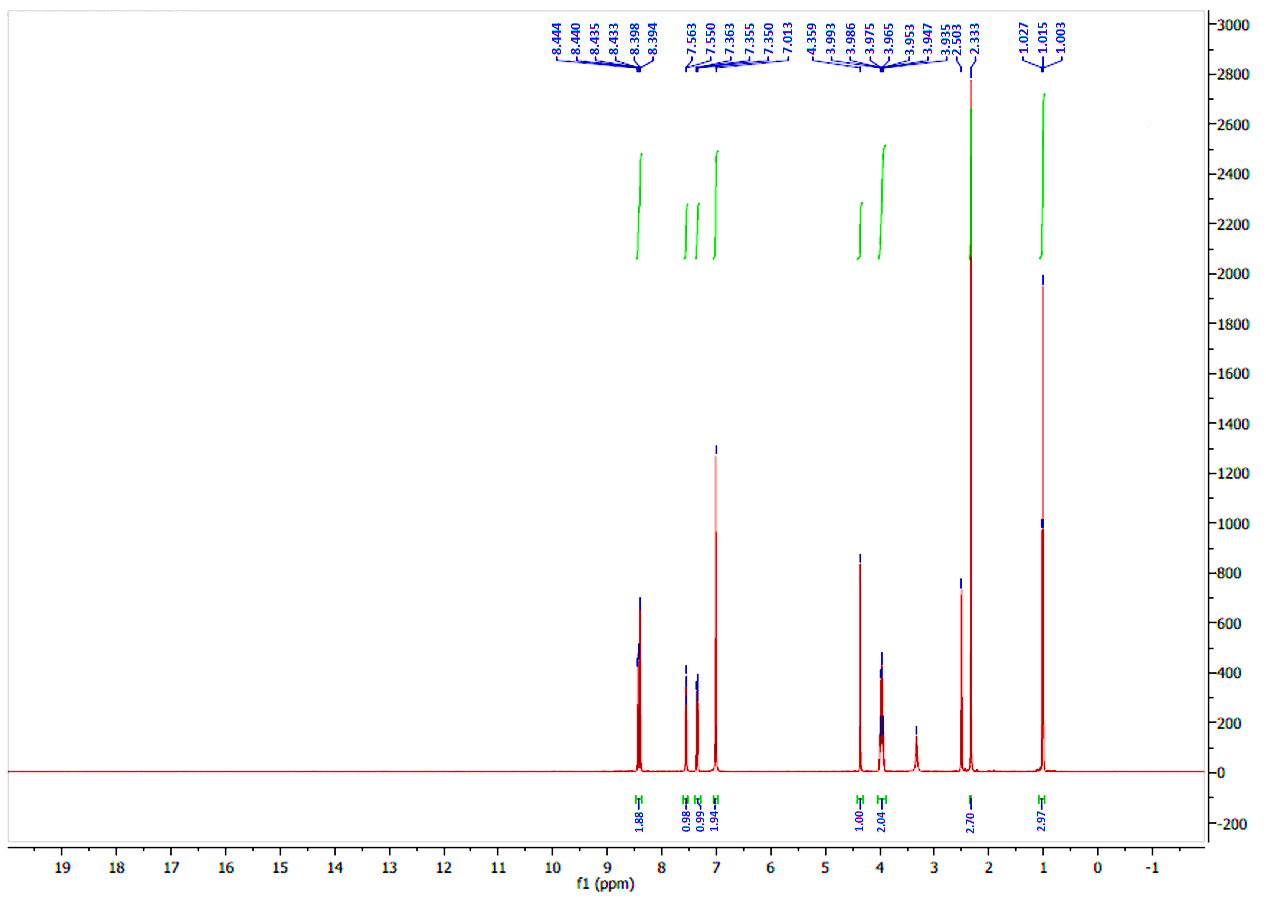


**Fig. 10.** ^1^H NMR spectrum of ethyl 6-amino-5-cyano-2-methyl-4-(pyridin-3-yl)-4H-pyran-3-carboxylate (**5j**).

4) **Ethyl 6-amino-5-cyano-2-methyl-4-(thiophen-2-yl)-4*H*-pyran-3-carboxylate (5k):** mp = 174 − 176 °C; Yield = 89 %; ^1^H NMR (500 MHz, DMSO‑*d_6_*), *δ* (ppm): 7.35 (1H, d, *J* = 4.8 Hz, Ar-H), 7.02 (2H, brs, NH_2_), 6.93 (1H, t, *J* = 4.2 Hz, Ar-H), 6.83 (1H, d, *J* = 3.0 Hz, Ar-H), 4.63 (1H, s, CH), 4.07 (2H, *J* = 7.2 Hz, CH_2_), 2.27 (3H, s, CH_3_), 1.14 (3H, t, *J* = 7.2 Hz, CH_3_).

*
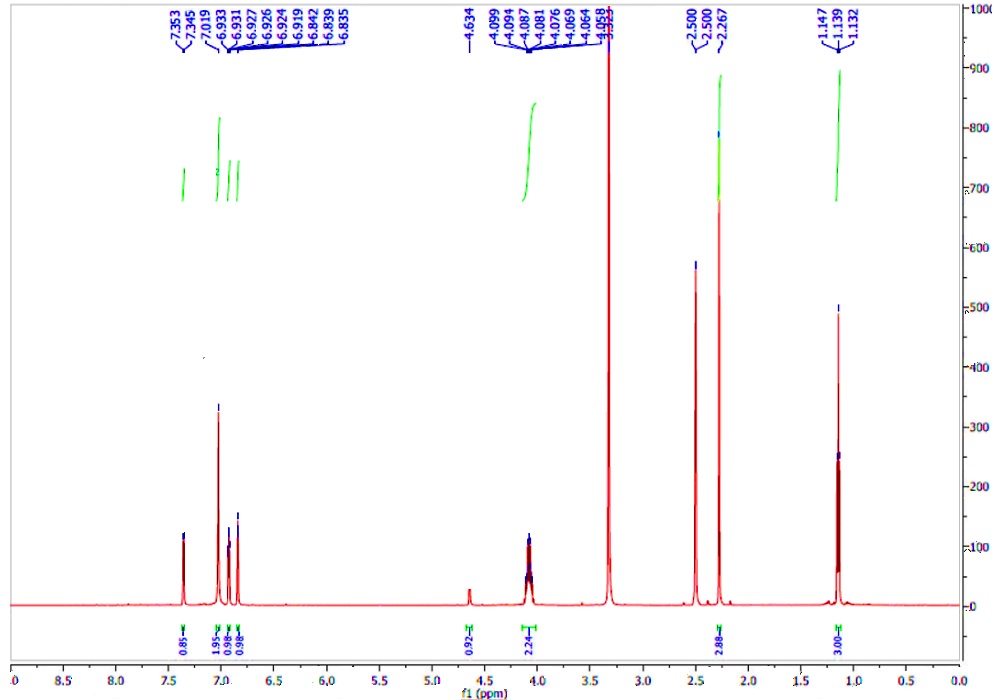
*

**Fig. 11.**^1^H NMR spectrum of ethyl 6-amino-5-cyano-2-methyl-4-(thiophen-2-yl)-4H-pyran-3-carboxylate (**5k**).
